# Bone metastatic carcinoma coexisting with plasma cell myeloma: a case report

**DOI:** 10.1186/s13256-022-03688-x

**Published:** 2022-12-23

**Authors:** Marwa Aloui, Sarra Ben Rejeb, Manel Boudokhan, Amen Dhaoui, Majdi Ben Romdhane, Syrine Belakhal

**Affiliations:** 1 Pathology Department, Security Forces Hospital, Marsa, Tunisia; 2 Medicine Department, Security Forces Hospital, Marsa, Tunisia; 3 Orthopedic Department, Security Forces Hospital, Marsa, Tunisia

**Keywords:** Plasma cell myeloma, Bone metastasis, Solid carcinoma, Synchronous malignancies, Osteolytic bone lesions

## Abstract

**Objective:**

Multiple myeloma is a clonal plasma cell proliferation often causing bone lytic lesions. It is sometimes challenging to differentiate these lytic lesions associated with multiple myeloma from bone destruction due to a metastasis. Although coexistence of solid tumors and plasma cell myeloma in one patient has been described, synchronous skeletal metastases from both neoplasms occurring in the same bone lesion is exceptional. Indeed, only one case has been reported in the literature.

**Case presentation:**

Herein, we report a case involving a 68-year-old Caucasian male patient admitted to our department for coronavirus disease 2019 infection with incidental finding of multiple lytic bone lesions during hospitalization. Laboratory tests revealed an increased immunoglobulin G kappa M protein and high levels of carbohydrate antigen 19-9. Bone marrow aspiration showed increased atypical plasma cells consistent with multiple myeloma. Percutaneous image-guided biopsy of one of the osteolytic lesions was performed. Pathological examination identified both plasma cell neoplasm and poorly differentiated metastatic carcinoma within the same bone lytic lesions.

**Conclusion:**

The present case raises awareness among clinicians and pathologists that clinical and radiologic suspicion of multiple myeloma may be within the spectrum of second primary malignancies.

## Introduction

The bone is one of the most common sites of metastases after the lung and the liver [1]. The type of lesions depends on the mechanism of interference and normal bone remodeling. The lesions may be osteolytic, osteoblastic, or mixed. In lytic skeletal lesions, destruction of normal bone is noted. Such lesions are mainly observed in multiple myeloma (MM) and solid cancer metastases, such as renal cell carcinoma, prostate carcinoma melanoma, non-small cell lung cancer, thyroid cancer, Langerhans cell histiocytosis, and the majority of breast cancers [2]. Although the clinical presentation and some radiological clues are potentially helpful in differentiating lytic lesions in MM from other cancer metastases, pathological examination with immunohistochemical staining remains necessary to clearly identify the histological subtypes of tumor cells.

Some reports have described the associations of MM with solid tumor cancers occurring in the same patient, and they investigated the challenging differential diagnosis of lytic lesions in such cases. However, coexisting skeletal lytic lesions of both malignancies in the same bone is extremely rare and, to the best of the authors’ knowledge, only one case has been reported in the literature [3].

Herein, we report another challenging diagnosis of synchronous osteolytic lesions involving MM and metastatic carcinoma.

## Case report

**Fig. 1:**
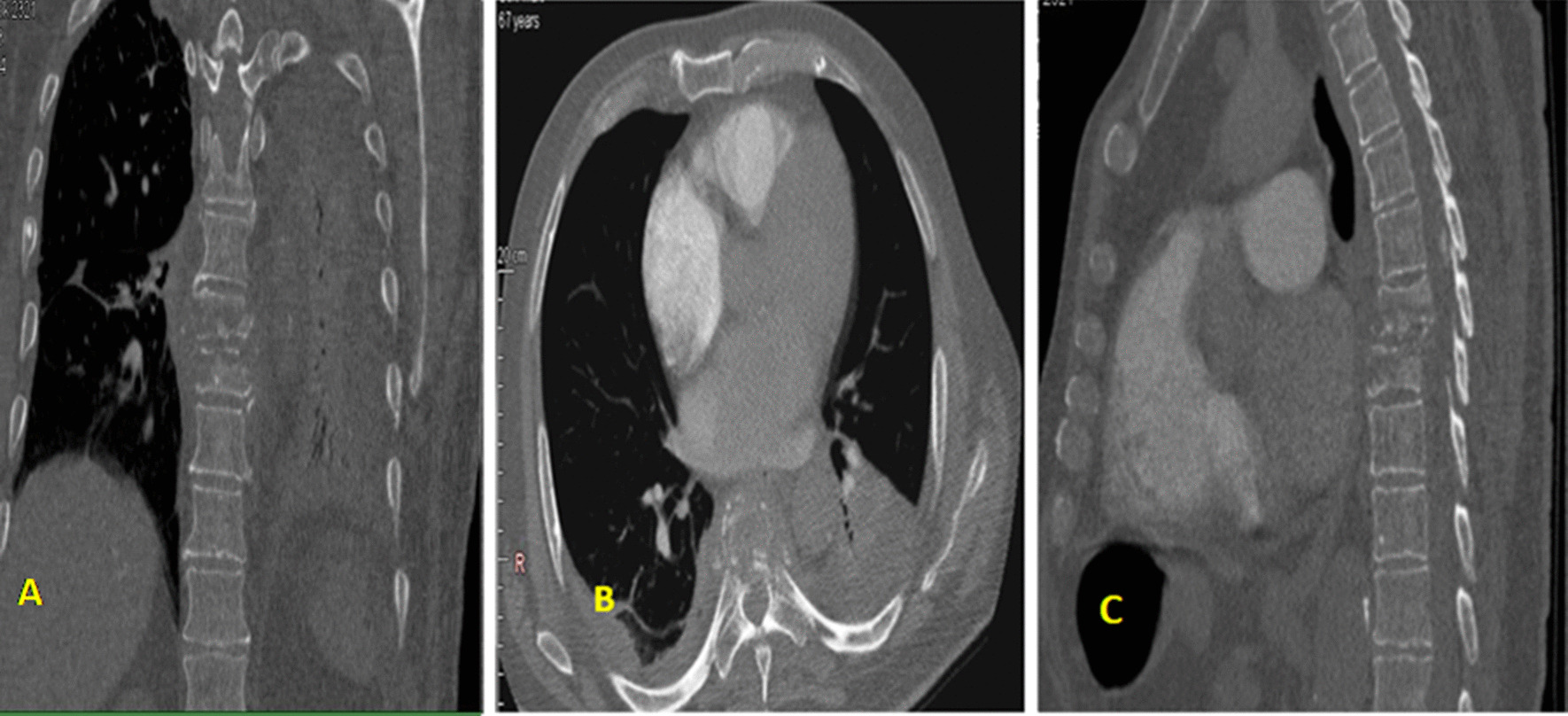
Compressive fracture of the D6–D7 vertebrae (**A**), Heterogeneous lytic appearance of D6–D7 vertebrae on axial (**B**) and sagittal (**C**) cross-sections, classified as 1c according to Lodwick classification

A 68-year-old Caucasian male patient with a medical history of prostate gland hyperplasia and infectious spondylodiscitis was admitted to our department for severe acute respiratory syndrome coronavirus 2 (SARS-CoV-2) infection. He was treated with oxygen therapy, antibiotics, and antithrombotic agents. On first laboratory tests, complete blood count revealed an hypochromic microcytic anemia [hemoglobin at 8.5 g/dl (13–17 g/dl)] and increased leucocytes [12,110/mm^2^ (4000–10,000/mm^2^)]; the renal function was normal [serum creatinine, 59 µmol/l (65–127 µmol/l)] and high levels of D-dimers were found [1990.42 ng/ml (< 500 ng/ml)], which may suggest an associated embolism. Hence, an iodine-injected thoracoabdominal computed tomography scan (CT) was performed to rule out a pulmonary embolism. It showed no embolus; however, it incidentally revealed osteolytic lesions in the second and ninth left ribs and the D6–D7 vertebrae, causing a compressive fracture at this level (Fig. 1). Retroperitoneal lymph node enlargement was also noted.

The renal function tests performed 48 hours after the iodine-injected CT scan revealed an increased serum creatinine at 148 µmol/l (65–127 µmol/l), indicating a contrast-induced acute kidney injury (a rise > 50% above the first value before the CT scan (59 µmol/l)); which made it unsafe to perform CT colonography with contrast to check the colon for possible signs of cancer.

**Fig. 2:**
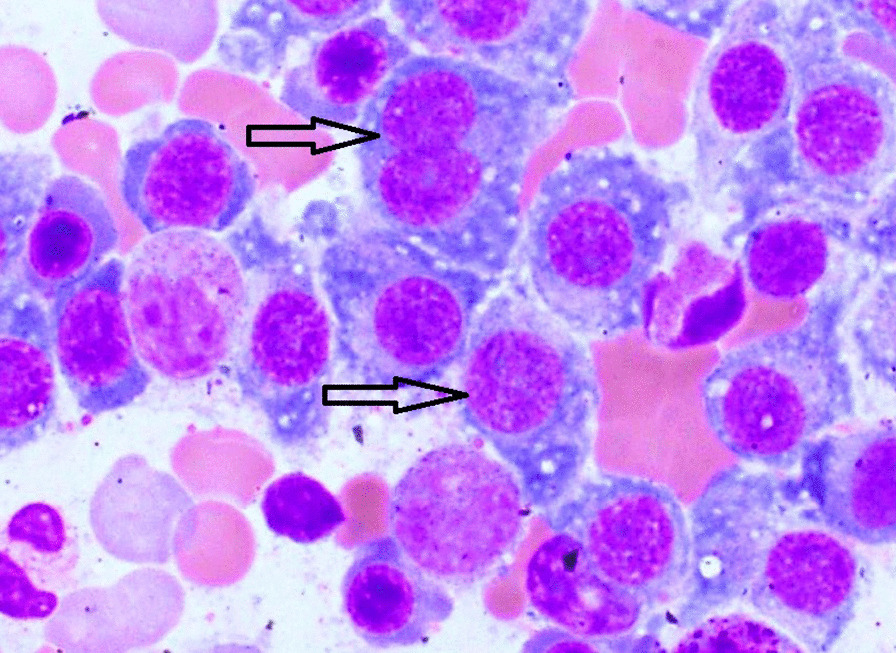
Bone marrow aspiration showing atypical mature and immature plasma cells (black arrows)

**Fig. 3:**
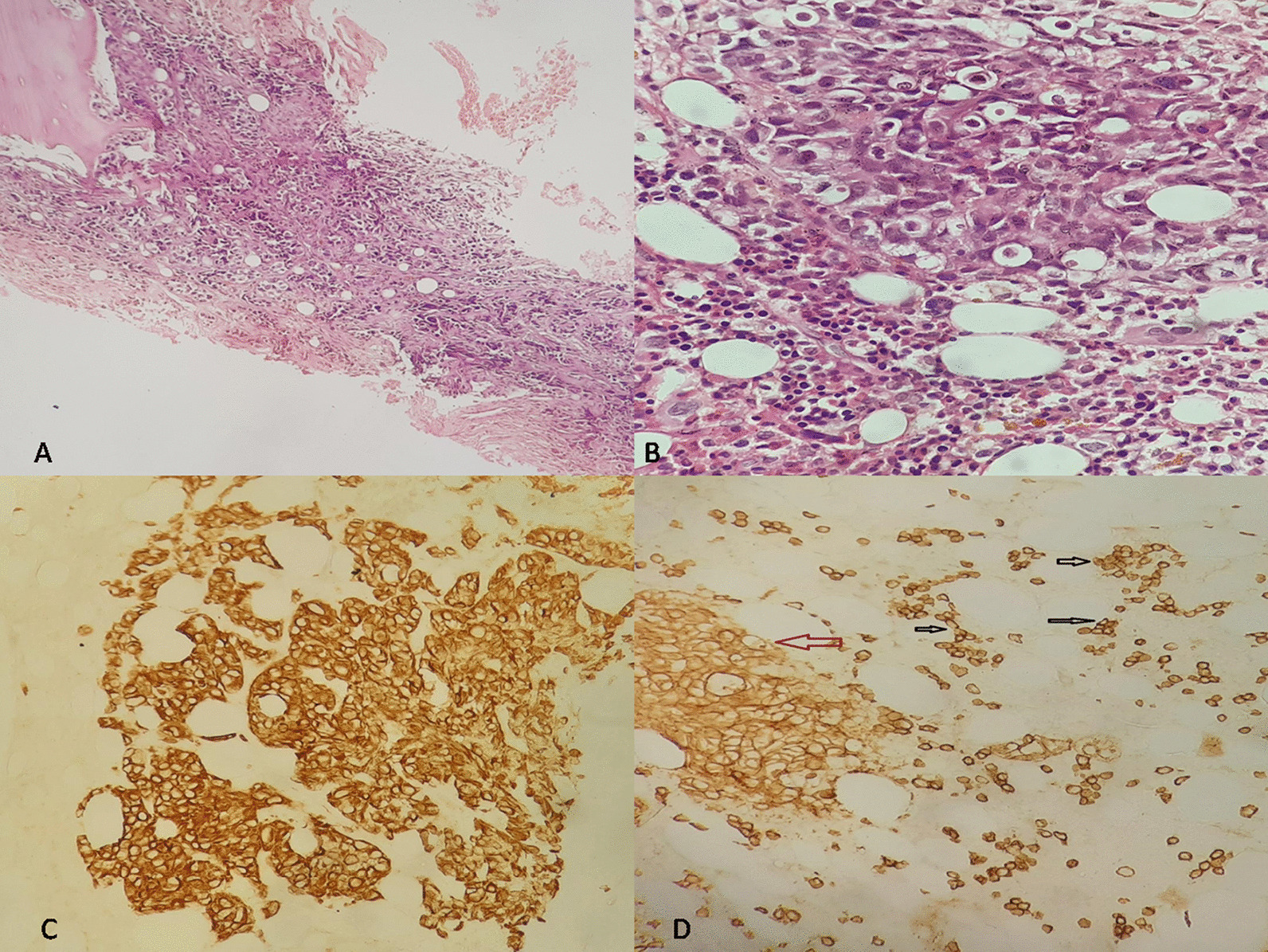
**A** Hematoxylin and eosin (HE) ×20: Bone marrow trephine section showing diffuse infiltration with nests and islands of carcinomatous-like cells set in a fibrous stroma. **B** HE ×40: Large carcinomatous tumor cells with atypical and hyperchromatic nuclei. **C** Immunohistochemistry (IHC) ×20: The carcinomatous component shows diffuse positive staining for CK7. **D** IHC ×10: CD138 showing positive staining for sparse small plasma cells. The red arrow points to the positive staining of the carcinomatous cells for CD138. Black arrows point to the positive staining of the plasma cells for CD138

**Fig. 4:**
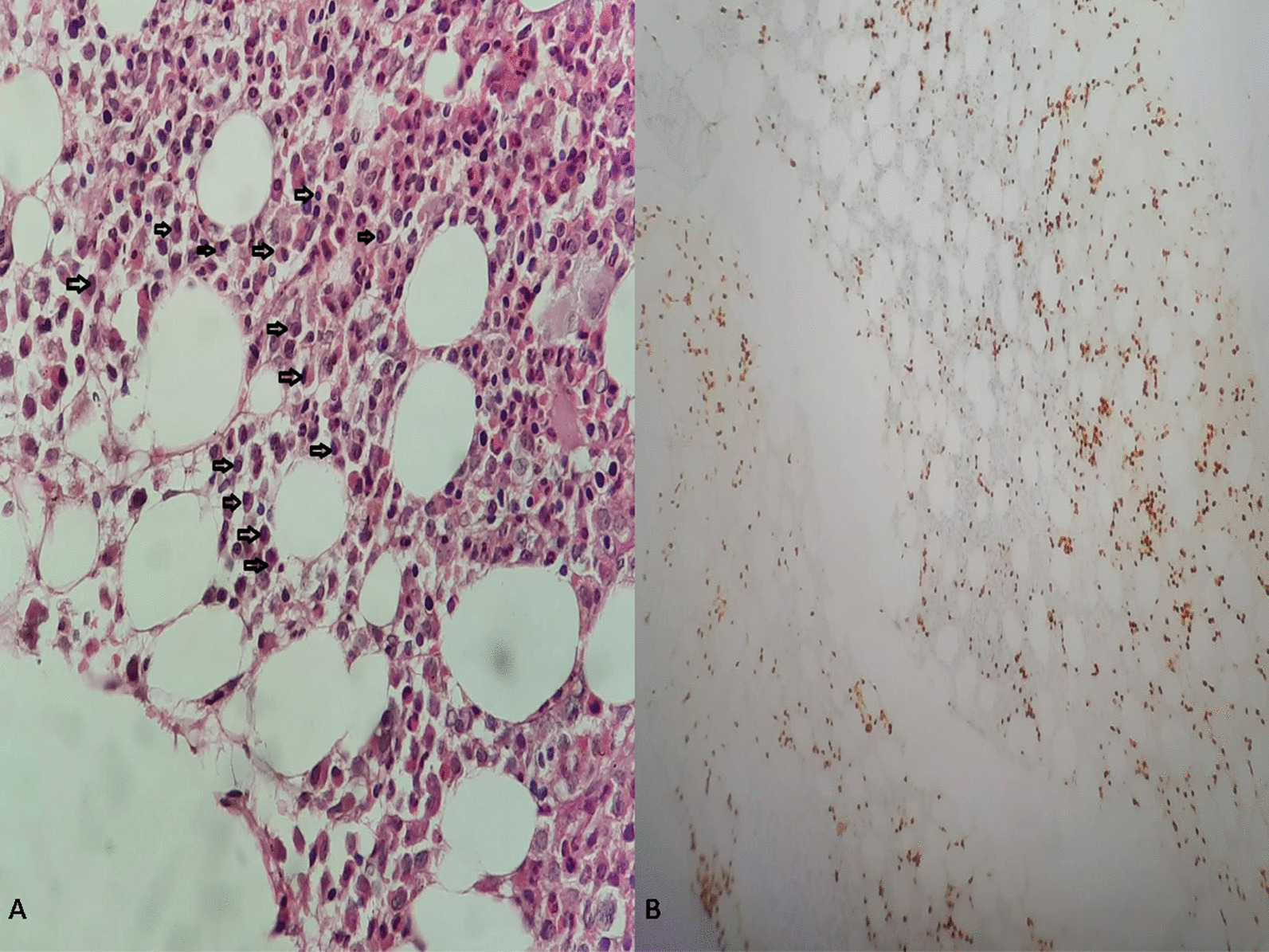
**A** HE ×20: Other areas of the bone marrow section showing increased plasma cells. **B** IHC ×10 MUM1 staining showing interstitial and nodular plasma cells

The skeletal radiography revealed multiple osteolytic lesions in the vertebrae body, iliac bone, and femur. Imaging features were highly suggestive of either MM or metastasis from a solid tumor. Additional laboratory tests revealed elevated carbohydrate antigen (CA) 19-9 [163.65 U/ml (< 37 U/ml)], hypercalcemia (2.80 mg/l), elevated creatinine [231 µmol/l (65–127 µmol/l)], hyperuricemia [15.1 mmol/l (2.8–7.2 mmol/l)], and hypoalbuminemia [21.4 g/l (35–52 g/l)]. Prostate specific antigen (PSA) [0.69 ng/ml (< 4 ng/ml)], α-fetoprotein (AFP) [0.98 U/ml (< 15 U/ml)], and carcinoembryonic antigen (CEA) [10.21 ng/ml (< 10 ng/ml)] were normal. Serum protein electrophoresis (ELP) revealed an increased immunoglobulin G (IgG) kappa M protein. Bone marrow aspiration showed an increased number of mature or immature atypical plasma cells (Fig. 2). These findings highly supported the diagnosis of MM, although the enlarged abdominal lymph nodes noted in the abdominal CT scan and the elevated CA 19-9 were suggestive of concomitant metastatic carcinoma. Percutaneous image-guided biopsy of the iliac lytic lesion was performed. On histological examination, the bone marrow trephine biopsy section showed variable features. In some areas, diffuse replacement of the marrow by dense fibrous tissue containing nests and islands of cohesive large cells with atypical and hyperchromatic nuclei was noted (Fig. 3-A, B). In other areas, interstitial and nodular infiltration of clusters of small plasmacytoid-like cells was observed. On immunohistochemical analysis, the epithelial-like large cells were positive for AE1/AE3, EMA, CK7, CK19, and CD138 (Fig. 3-C, D). They showed no staining for MUM1, CK20, TTF1, and PSA. The plasmacytoid-like cells were positive for CD138 and MUM1, and they were negative for all epithelial and other lymphoid markers (Fig. 4). The plasma cell population in the bone marrow was assessed at 20–25%. On the basis of these histological and immunohistochemical findings, diagnosis of poorly differentiated metastatic carcinoma of the bone marrow was made. However, given the increased plasma cell population on bone marrow aspiration and biopsy, diagnosis of synchronous plasma cell myeloma was retained.

The patient developed renal failure and metabolic disorders secondary to coronavirus disease 2019 (COVID-19) infection. He passed away within a few days. The primary tumor site was not identified.

## Discussion

Multiple myeloma is a clonal proliferation in the bone marrow of neoplastic plasma cells [4]. To make diagnosis of MM, two of the following criteria must be met: (1) more than 10% of plasma cells in bone marrow aspirate, (2) paraprotein in serum or urine, and (3) osteolytic bone lesions or osteoporosis [4]. However, these spots of bone destruction may also be seen in other conditions, especially in osseous metastases from solid cancers. Some radiological particularities that could be helpful for differential diagnosis have been reported by some authors. Indeed, bone destruction caused by cancer metastasis mostly involves the vertebral pedicles, whereas MM lesions are more commonly seen on the vertebral bodies and frequently involve the mandible and the distal axial skeleton [5]. Moreover, the lytic bone lesions suggestive of MM are characteristically sharply defined and punched out with endosteal scalloping leading to a very typical “raindrop skull” appearance. In another study, Uygar *et al*. [6] compared the CT features of MM and osteolytic metastatic bone lesions and concluded that the presence of high density, lesional homogeneity, perilesional sclerosis, and marginal features could be used to distinguish metastatic from MM lesions. According to Lee *et al*. [7] magnetic resonance imaging (MRI) is useful in distinguishing MM from metastasis involving the spine; indeed, the salt and pepper infiltration pattern, the presence of more than five lesions within one vertebra, and the involvement of more than three consecutive vertebrae are highly suggestive of MM. However, they reported no significant differences in signal intensities or enhancement patterns. In conclusion, the authors argue for a high overlap between bone lytic lesions attributed to MM and those related to cancer metastases on radiologic investigations. In such a context, ^18^F-fluorodeoxyglucose (FDG)-positron emission tomography (PET)-CT could be helpful in detecting not only bone metastatic lesions, but also primary solid tumors. In addition, some laboratory biomarkers are associated with solid cancers and could be an argument favoring cancers metastasis in difficult cases. Elevated CA 19-9, CEA, and AFP are more commonly associated with a gastrointestinal tumor; high levels of prostate-specific antigen (PSA) indicate prostate cancer. However, only biopsy with pathological examination could confirm the proper diagnosis.

In this case report, although lytic multiple lesions were first thought to be related to MM, the unusual findings of enlarged lymph nodes and elevated CA 19-9 made it necessary to consider the possibility of synchronous MM and solid tumor. Many papers have described synchronous solid tumors and MM mainly discovered on whole body scan. In this context, it is highly challenging to relate bone lytic lesions to either MM or tumor metastasis [3, 8–13].

However, to the best of the authors’ knowledge, only the case reported by Herrera *et al*. [3], involving MM and metastatic carcinoma affecting the same bone lesion, is similar to the present case. Pathological and immunohistochemical examinations are necessary to identify both components. Although the morphological examination could clearly distinguish carcinomatous cells from plasma cell proliferation, immunohistochemistry (IHC) is necessary to detect the tumor cells associated antigens. On IHC, pan-cytokeratin (AE1/AE3) and EMA positivity are consistent with the epithelial nature of the tumor cells. Other immunohistochemical markers, such as CK7, CK20, and organ-specific markers, are helpful to assess the primary origin of the disease. CD138 is a monoclonal anti‐syndecan‐1 antibody often used to identify plasma cells in the bone marrow of patients with MM. However, several carcinomas may also express CD138, including prostate, colon, renal cell, and hepatocellular carcinomas.

Distinguishing epithelial cells from plasma cells can easily be made based on microscopic examination. Indeed, epithelial cells tend to be larger with variable amounts of cytoplasm and marked nuclear atypia, and, as in the present case, mostly arranged on islands, nests, or glands with marked fibrous stromal reaction. However, plasma cells are smaller, with few amounts of cytoplasm, and they are mostly sparse. Moreover, MUM1, also known as interferon regulatory factor 4 (IRF4), is another interesting highly specific marker for normal and neoplastic plasma cells. It is classically negative on epithelial cells. In the present case, tumor cells showed (CK7^+^, CK20_−_, CK19^+^, CD138^+^) immunohistochemical profile. As presented above, CD138 is not specific and it may be expressed in various primary carcinomas. However, the most contributive marker is CK7. Hence, the immunohistochemical findings in the present case may be mainly seen in renal cell carcinomas, breast carcinomas, papillary thyroid carcinomas, lung adenocarcinomas, and biliary and pancreatic carcinomas [14]. Less frequently, they may be seen in urothelial bladder carcinomas, gastric adenocarcinomas, and squamous cell carcinomas. IHC studies should therefore be always ordered in correlation with the clinical, radiological, and pathological findings. Indeed, immunohistochemical markers are not always specific and aberrant expression may be seen in various tumor cancers.

The etiopathogenesis of this reported association is not yet well established. Bone marrow reactive plasmacytosis occurs in a variety of diseases, including carcinomas, lymphoproliferative disorders, and inflammatory conditions, and hence could be a precursor for MM. Another possible theory is the disruption of the immune system secondary to the development of multiple myeloma, which could impair the immune surveillance and resistance to cancer cells [11].

## Conclusions

The authors reported an extremely rare association of bone metastatic carcinoma and plasma cell myeloma arising in the same bone. This paper contributes to increasing awareness among clinicians and pathologists that the clinical and radiologic suspicion of MM may be found within the spectrum of a second primary malignancy. Clinicians and pathologists must bear in mind that MM can occur synchronously or metachronously with other malignancies, especially in the presence of other coexisting soft tissue or lymph node masses.

Further studies are needed to investigate the etiopathogenesis of these associations.

## Data Availability

Not applicable.
